# Comparative study of the interactions between fungal transcription factor nuclear localization sequences with mammalian and fungal importin-alpha

**DOI:** 10.1038/s41598-020-58316-9

**Published:** 2020-01-29

**Authors:** Natália E. Bernardes, Cintia A. Fukuda, Tainá D. da Silva, Hamine C. de Oliveira, Andrea C. de Barros, Thiago R. Dreyer, Maria Célia Bertolini, Marcos R. M. Fontes

**Affiliations:** 1 Departamento de Física e Biofísica, Instituto de Biociências, Universidade Estadual Paulista (UNESP), Botucatu, São Paulo Brazil; 2 Departamento de Bioquímica e Tecnologia Química, Instituto de Química, Universidade Estadual Paulista (UNESP), Araraquara, São Paulo Brazil

**Keywords:** Carrier proteins, Nucleoproteins, X-ray crystallography, Molecular biophysics

## Abstract

Importin-α (Impα) is an adaptor protein that binds to cargo proteins (containing Nuclear Localization Sequences - NLSs), for their translocation to the nucleus. The specificities of the Impα/NLS interactions have been studied, since these features could be used as important tools to find potential NLSs in nuclear proteins or even for the development of targets to inhibit nuclear import or to design peptides for drug delivery. Few structural studies have compared different Impα variants from the same organism or Impα of different organisms. Previously, we investigated nuclear transport of transcription factors with *Neurospora crassa* Impα (NcImpα). Herein, NIT-2 and PAC-3 transcription factors NLSs were studied in complex with *Mus musculus* Impα (MmImpα). Calorimetric assays demonstrated that the PAC-3 NLS peptide interacts with both Impα proteins with approximately the same affinity. The NIT-2 NLS sequence binds with high affinity to the Impα major binding site from both organisms, but its binding to minor binding sites reveals interesting differences due to the presence of additional interactions of NIT-2-NLS with MmImpα. These findings, together with previous results with Impα from other organisms, indicate that the differential affinity of NLSs to minor binding sites may be also responsible for the selectivity of some cargo proteins recognition and transport.

## Introduction

Nucleocytoplasmic protein trafficking regulation between cell compartments is a fundamental biological process for eukaryotic organisms. The translocation of proteins across the nuclear envelope occurs through nuclear pore complexes (NPC) which, in most cases, it is an active carrier-mediated transport process^1,2^. This mechanism requires additional carrier proteins or transport factors that generally belong to the β-karyopherin superfamily and specific nuclear targeting signals. The best-characterized signals are known as nuclear localization sequences (NLS), which are recognized by the importin-α protein (Impα). Impα is an adaptor protein that links the cargo protein to a carrier protein (importin-β; Impβ) that, through transient interactions between Impβ and NPC proteins, translocates the Impα/Impβ/cargo protein complex to the cell nucleus. This process is known as the classical nuclear import pathway and is probably the most extensively used and heavily researched nuclear import mechanism^3–5^.

The classical NLS (cNLS) is characterized by one or two amino acid basic clusters and is defined as monopartite or bipartite. Consensus sequences for these two cNLS were proposed and correspond to K(K/R)X(K/R) and KRX_10–12_K(K/R)X(K/R) (where X corresponds to any residue, but positively charged amino acids are preferred at this position and hydrophobic ones are also acceptable), respectively^4,6–9^. These sequences bind to Impα through hydrophobic and polar contacts with two binding sites, known as major and minor binding sites. Bipartite NLSs interact with both binding sites, and monopartite NLSs preferentially bind to the major site^4,6–8,10^. In addition, a random peptide library studied with Impα suggested six classes of NLSs, including two types of non classical NLSs: ‘plant-specific’ NLSs and ‘minor site-specific’ NLSs^11^.

Crystal structures of Impα from different organisms have been determined: *Homo sapiens*^9,12–21^, *Mus musculus*^4,5,7,8,22–35^, *Saccharomyces cerevisiae*^36–38^, *Oryza sativa*^33,39^, *Arabidopsis thaliana*^40^ and *Neurospora crassa*^41^. In some organisms, 12-several Impα variants exist. For example, *H. sapiens* and *M. musculus* have seven and six Impα isoforms identified, respectively, and each one is related to specific stages of development or is tissue-specific^15,42^. Metazoan paralogues can be divided into three clades (α1, α2 and α3) and Impα from Viridiplantae and Fungi belong to the α1-like clade^43–45^. Impα from *S. cerevisiae*^36–38^, *O. sativa*^33,39^, *A. thaliana*^40^ and *N. crassa*^41^ belong to the α1-like clade. Members of different subfamilies share approximately 50% sequence identity, whereas within a subfamily, the identities are >80%^43^. The structure and recognition mechanism are highly conserved among Impα proteins from different species^8,41^, but the existence of multiple Impα variants suggests that characteristics in each isoform allow them to selectively recognize different NLSs. Recognition of NLSs by more than one Impα isoform is related to the evolutionary history of those proteins and may also be critical for pathogenic organisms that use the host machinery to transport exogenous proteins into the nucleus of host cells as part of an infectious process^46–50^.

Structural studies with *O. sativa* Impα (OsImpα)^33,39^ identified NLSs that bind preferentially to the minor binding site. Likewise, a structural and calorimetric study with *N. crassa* Impα (NcImpα)^41^ and simian virus SV40 TAg NLS resulted in higher affinity of the NLS to the minor site for *N. crassa* Impα than for *M. musculus* Impα (MmImpα).

Previously, we investigated nuclear transport of NIT-2, a GATA transcription factor that plays a fundamental role in the regulation of nitrogen metabolism in *N. crassa*, using a combination of biochemical, cellular, biophysical and crystallographic methods^51^. The nuclear translocation of NIT-2 was studied using HeLa cells. This study showed that the NIT-2 NLS (^915^TISSKRQRRHSKS^927^) was recognized by NcImpα and that its transport occurred via the classical import pathway. The crystal structure of the NcImpα/NIT-2 NLS complex was solved, showing that the NLS peptide was bound to the major and minor NLS-binding sites of NcImpα, but its binding at the major binding site plays a major role. Indeed, the interaction between the NcImpα and the NIT-2 NLS was quantified with calorimetric assays, leading to the observation that the peptide bound to two sites with different affinities, which is typical of a monopartite NLS sequence.

Analogously, we also investigated nuclear transport of PAC-3, a transcription factor that belongs to the C_2_H_2_ zinc finger family and related to the alkaline pH stress response in *N. crassa*^52^. We demonstrate that PAC-3 preferentially localizes in the nucleus at alkaline pH stress and that the translocation may require NcImpα, since the putative PAC-3 nuclear localization signal (NLS) has a strong *in vitro* affinity with NcImpα using calorimetric assays.

In the present work, both previously studied NLSs from *N. crassa* transcription factors (NIT-2 and PAC-3) were cocrystallized with mammalian Impα, and their structures were solved. Isothermal Titration Calorimetry assays were performed to determine their dissociation constants and thermodynamic values. As a result of this study, we were able to compare, for the first time, the binding mode and affinity of fungus-encoded NLSs with Impα from different clades (α1 *versus* α1-like - fungus *versus* mammalian). Thus, these structural and calorimetric analyses were able to shade light in the nuclear transport of exogenous proteins that use the host machinery as part of an infectious process for pathogenic organisms.

## Results

### Crystallographic structures of MmImpα/NIT-2-NLS and MmImpα/PAC-3-NLS

**Figure 1:**
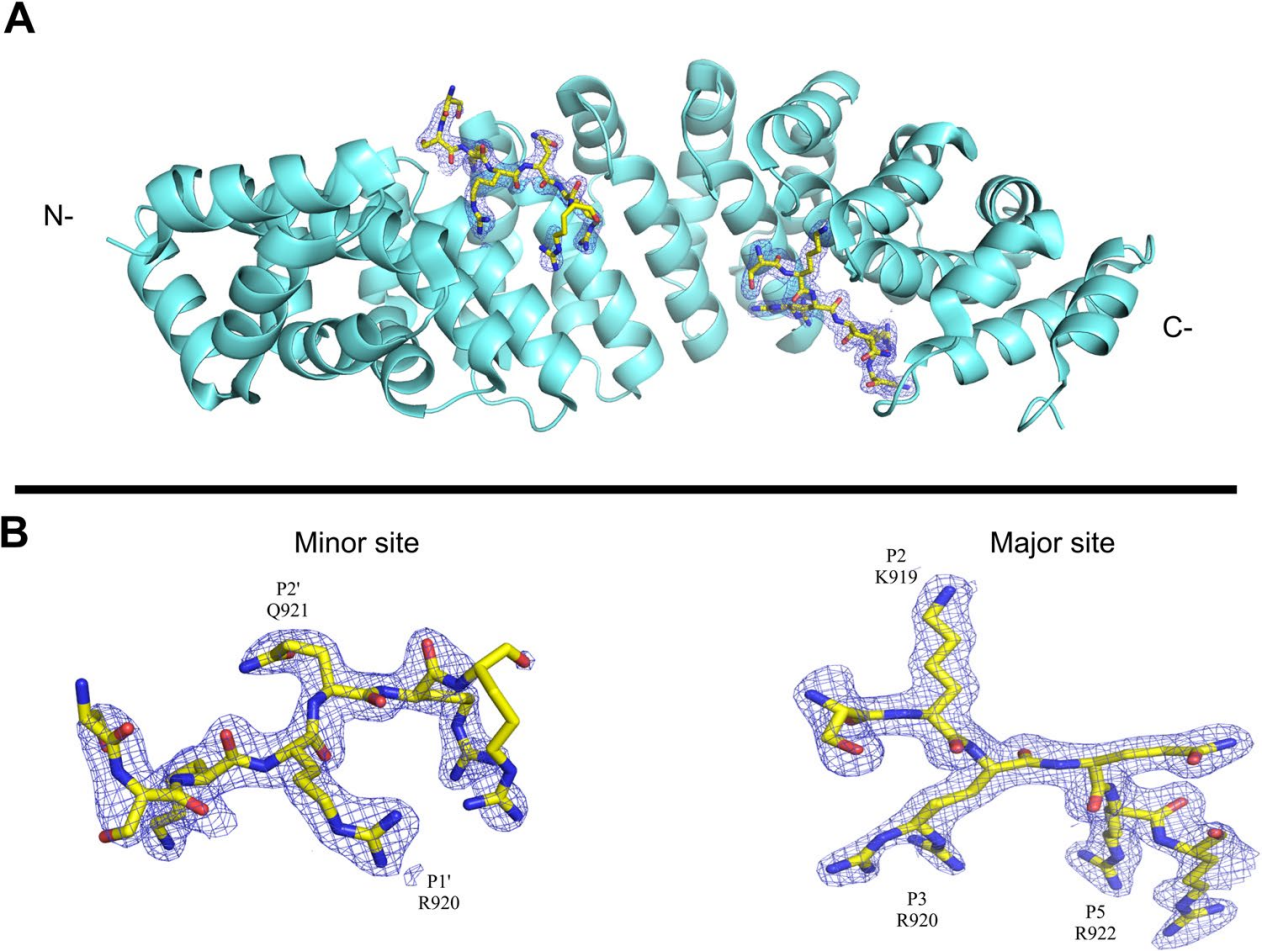
Cartoon representation of the MmImpα/NIT-2 NLS crystal structure. (**A**) MmImpα protein is shown in cartoon representation and the NIT-2 NLS peptide at the major and minor binding sites are shown in the stick representation. (**B**) Electron density map (coefficients 2|F_obs_|-|F_calc_|) corresponding to NIT2 NLS peptide at the major and minor site regions of Impα are contoured at 1.2 s.d. Some peptide residues are labeled at their corresponding binding positions. This figure was generated using PyMOL v.1.8.6^64^ program.

**Figure 2:**
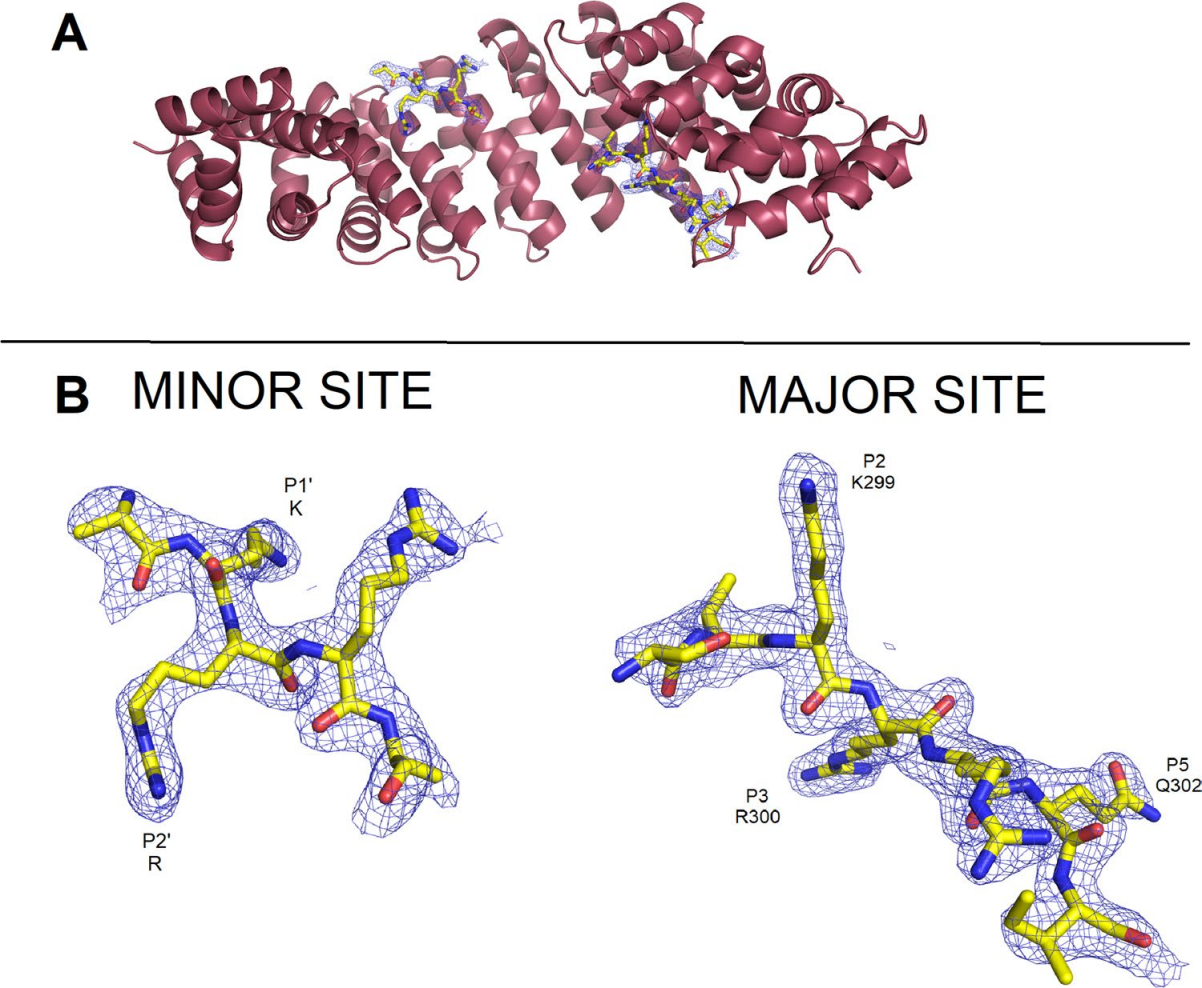
Cartoon representation of the MmImpα/PAC-3 NLS crystal structure. (**A**) MmImpα protein is shown in cartoon representation and the PAC-3 NLS peptide at the major and minor binding sites are shown in the stick representation. (**B**) Electron density map (coefficients 2|F_obs_|-|F_calc_|) corresponding to PAC-3 NLS peptide at the major and minor site regions of Impα are contoured at 1.2 s.d. Some peptide residues are labeled at their corresponding binding positions. This figure was generated using PyMOL v.1.8.6^64^ program.

NIT-2 NLS (^915^TISSKRQRRHSKS^927^) and PAC-3 NLS (^281^FDARKRQFDDLNDFFGSVKRRQIN^304^) peptides, corresponding to regions of *N. crassa* NIT-2 and PAC-3 transcription factors, were cocrystallized with N-terminally truncated *M. musculus* Impα lacking residues 1–69, variant α2 (MmImpα, UniProtKB: P52293). This truncated region is responsible for the autoinhibition of the Impα^22^. The crystal structures of MmImpα complexed to NIT-2-NLS (MmImpα/NIT-2-NLS) and MmImpα complexed to PAC-3-NLS (MmImpα/PAC-3-NLS) were solved at 2.15 and 1.99 Å, respectively (Table 1). The analysis of both MmImpα/NIT-2-NLS and MmImpα/PAC-3-NLS structures showed electron densities corresponding to fragments of the peptides in two different regions of the proteins, known as major and minor binding sites (Figs. 1 and 2, respectively). No electron density was found in the linker region between the major and minor binding sites. Similar to other Impα structures, the major binding site is located at armadillo (ARM) repeats 2–4, and the minor site is located at ARM repeats 6–8. Coordinates and structure factors have been deposited in the PDB under accession codes 6P6A (MmImpα/NIT-2-NLS) and 6P6E (MmImpα/PAC-3-NLS).

**Table 1:** Crystallographic data for MmImpα/NIT-2 NLS and MmImpα/PAC-3 NLS complexes. Numbers in parenthesis correspond to the highest resolution data.

	MmImpα/NIT2	MmImpα/PAC3
Unit cell parameters (Å)	a = 78.7 b = 90.2 c = 99.2	a = 78.5 b = 90.5 c = 99.7
Space group	P2_1_2_1_2_1_	P2_1_2_1_2_1_
Resolution (Å)	36.57–2.15 (2.23–2.15)	43.67–1.99 (2.07–1.99)
Unique reflections	38,951 (3788)	49,032 (4799)
Completeness (%)	99.82 (99.11)	99.81 (99.78)
R_merge_^a^	0.116 (0.36)	0.118 (0.46)
I/σ (I)	21.26 (2.28)	18.29 (0.97)
Multiplicity	12.7 (11.6)	13.0 (12.5)
CC ½	0.999 (0.819)	0.999 (0.469)
Total reflections	49,5854	63,7123
R_work_^b^ (%)	16.56	17.49
R_free_^c^ (%)	19.62	19.66
Number of non-H atoms:		
Protein	3,278	3,333
Peptide	128	120
Solvent	290	314
Average B factor (Å^2^)	44.12	47.35
RMS (bonds)	0.008	0.004
RMS (angles)	1.22	0.99
Clashcore	5.04	2.82
Ramachandran plot:		
Residues in most favored regions (dissallowed) (%)	98.38 (0.0)	98.98 (0.00)

^a^Rmerge= Σhkl(Σi(|Ihkl,i-<Ihkl>|))/Σhkl,i<Ihkl> at where I hkl,i é is the intensity of each individual measure of the reflection with Miller indices h, k and l, and <Ihkl> is the average intensity of that reflection. Calculated for I> −3% (I) (OTWINOWSKI Z *et al*., 1997).

^b^Rcryst = hkl(||Fobshkl|-|Fcalchkl||)/|Fobshkl|, at where |Fobshkl| and |Fcalchkl| are the amplitudes of observed and calculated structure factors.

^c^Rfree is equivalent to Rcryst, but calculated based on 5% of the total reflection.

### Binding of NIT-2 NLS to the MmImpα

**Figure 3:**
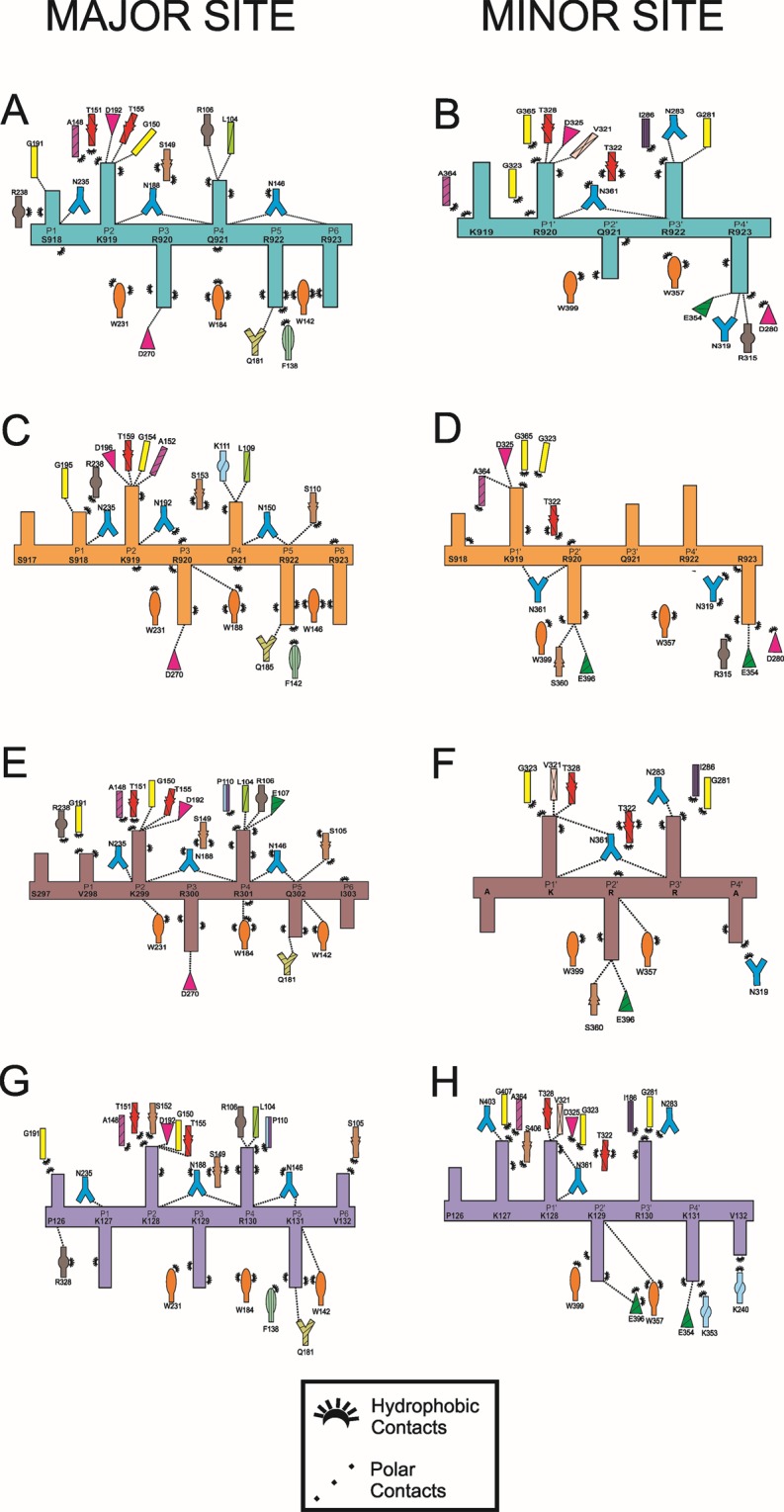
Schematic diagram of the interactions between the NIT-2 NLS, PAC-3 NLS and SV40 NLS peptides and the minor and major binding sites of MmImpα and NcImpα. (**A**) MmImpα/NIT-2 NLS - major binding site. (**B**) MmImpα/NIT-2 NLS - minor binding site. (**C**) NcImpα/NIT-2 NLS^51^- major binding site. (**D**) NcImpα/NIT-2 NLS - minor binding site. (**E**) MmImpα/PAC-3 NLS - major binding site. (**F**) MmImpα/PAC-3 NLS - minor binding site. (**G**) MmImpα/SV40 NLS - major binding site. (**H**) MmImpα/SV40 NLS - minor binding site. The peptide backbones are drawn in cyan (MmImpα/NIT-2 NLS), orange (NcImpα/NIT-2 NLS), brown (MmImpα/PAC-3 NLS) or violet (MmImpα/SV40 NLS) with the residues identified by the one-letter code. The Impα side-chain residues interacting with the peptide are indicated with their names and different colors. The polar contacts are shown with dashed lines, and the hydrophobic contacts are indicated by arcs with radiating spokes. This figure was generated using PyMOL v.1.8.6^64^ program.

The crystal structure of MmImpα/NIT-2 NLS presented two fragments of NIT-2 NLS peptide bound to major and minor sites, which is similar to several monopartite NLS-MmImpα structures^7,53^. Electron density is present for seven peptide residues (^917^SSKRQRR^923^) at the major NLS-binding site, bound at positions P0-P6 of MmImpα. The peptide presents an average B-factor of 56.6 Å^2^ (the average B-factor for the entire Impα is 43.4 Å^2^) (Fig. 1). The residues bound to the core of the major NLS-binding site (residues 919–922; positions P2–P5) have average B-factors (53.6 Å^2^) comparable to Impα. All these residues (919–922) present charged interactions between their side-chains and Impα side-chain residues (Fig. 3A).

**Figure 4:**
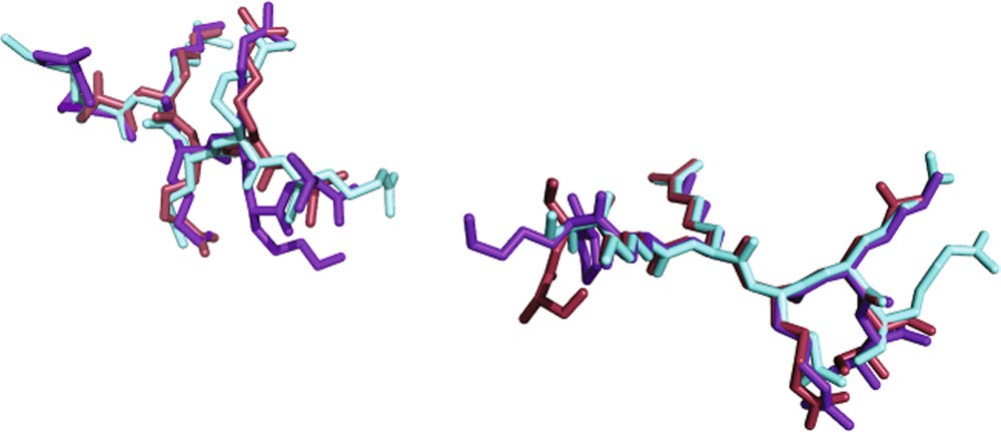
Comparison of NLS peptides at the major and minor NLS binding sites of MmImpα. NIT-2 NLS (cyan), PAC-3 NLS (brown) and SV40 NLS (violet)^7^. Positions binding to the major (P_1_-P_5_) and minor binding sites (P_1_′-P_4_′) are identified along the chains. This figure was generated using PyMOL v.1.8.6^64^ program.

Electron density is also present for six peptide residues (^918^SKRQRR^923^) at the minor NLS-binding site, bound at the positions P0′-P5′ of the MmImpα. The peptide presented an average B-factor of 43.0 Å^2^ (the average B-factor for entire Impα is 43.4 Å^2^) (Fig. 1). The residues bound to the core of the minor NLS-binding site (residues 919–922; positions P1′–P4′) have lower average B-factors (36.6 Å^2^) compared to Impα. K919, R920 and R923 residues (positions P1′, P2′ and P5′) present charged interactions between their side-chains and Impα side-chain residues (Fig. 3A). The superposition of Cα atoms between NIT-2 and SV40 NLS peptides yields an RMSD of 1.02 Å for the major binding site (positions P1–P5) and 0.57 Å for the minor binding site (positions P1′–P4′). Interestingly, these values are higher than in previous comparisons with SV40 NLS^53^, reflecting the high structural variability at the N- and C-termini of both NIT-2 NLS peptides in comparison with SV40 NLS peptide (Fig. 4).

### Binding of PAC-3 NLS to the MmImpα

The crystal structure of MmImpα/PAC-3 NLS presented two fragments of PAC-3 NLS peptide bound to major and minor sites. Electron density is present for seven peptide residues (^297^SVKRRQI^303^) at the major NLS-binding site, bound at positions P0-P6 of MmImpα. The peptide presents an average B-factor of 53.0 Å^2^ (the average B-factor for entire Impα is 46.8 Å^2^) (Fig. 2). The residues bound to the core of the major NLS-binding site (residues 299–302; positions P2–P5) have lower average B-factors (45.7 Å^2^) compared to Impα. All these residues (299–302) present charged interactions between their side-chains and Impα side-chain residues (Fig. 3B).

Electron density is present for five peptide residues (^298^AKRRA^302^) at the minor NLS-binding site, bound at the positions P0′-P4′ of the MmImpα. The KRR residues were modeled at the positions P1′-P3′ and Ala residues were modeled at positions P0′ and P4′ due to the lack of electron densities for their side chains (Fig. 2). The residues bound to the core of the minor NLS-binding site (residues KRR; positions P1′–P3′) have higher average B-factors (61.0 Å^2^) compared to Impα. These residues (positions P1′–P3′) present interactions between their side-chains and Impα side-chain residues (Fig. 3B). The superposition of Cα atoms between PAC-3 and SV40 NLS peptides yields an RMSD of 0.78 Å for the major binding site (positions P1–P5) and 0.31 Å for the minor binding site (positions P1′–P4′). Similar to NIT-2 NLS and SV40 NLS comparison (previous section), the comparison between PAC-3 and SV40 NLS peptides yielded higher than other equivalent comparisons^53^ with SV40 NLS. As seen in Fig. 4, NIT-2, PAC-3 and SV40 NLS in both sites display high structural variability at the N- and C-termini.

### Comparison between MmImpα structures and MmImpα/NIT-2 NLS and NcImpα/NIT-2 NLS structures

The superposition of the C_α_ atoms between the MmImpα/NIT-2 NLS and other MmImpα complexes (MmImpα/SART3 NLS, PDB ID 5CTT^54^, MmImpα/53BP1 NLS, PDB ID 6IUA^55^, MmImpα/Ku70 NLS, PDB ID 3RZX^32^, MmImpα/MLH1 NLS, PDB ID 5U5P^56^, MmImpα/XPG NLS, PDB ID 5EKF^53^ and MmImpα/P4(R) and P4(M) NLSs, PDB ID: 5KLR, 5KLT^9^) resulted in an average RMSD of 0.3 Å. This low value reflects the high structural conservation of the MmImpα, which is independent of the NLS peptide bound to them. In contrast, a similar superposition between MmImpα/NIT-2 NLS and NcImpα/NIT-2 NLS resulted an RMSD of 5.07 Å. This RMSD difference is the result of a more concave structure of the NcImpα compared to MmImpα, as previously observed^41^, which belong to different Impα clades.

Interestingly, despite the structural differences between NcImpα and MmImpα structures, the NIT-2 NLS peptide binds to NLS-binding sites with the exact same residues at each position of MmImpα and NcImpα (Fig. 3). The superposition of Cα atoms between NIT-2 NLSs from MmImpα and NcImpα structures yields an RMSD of 0.17 Å for the major binding site (positions P1–P5) and 0.51 Å for the minor binding site (positions P1′–P4′). The comparison of the NIT-2-NLS binding at the major site of MmImpα and NcImpα reveals that the contacts are very conserved, with the equivalent residues of both Impα proteins making contacts with NIT-2 NLS peptides. The same comparison for the minor binding site reveals interesting differences related to positions P3′ and P4′. While NIT-2 NLS interacts with N283 and G281 at position P3′ and with E354 N319 and R315 at position P4′ from MmImpα, no important interaction is observed between the NIT-2 NLS side-chain at positions P3′ and P4′ and NcImpα (Fig. 3). The structural data for the major and minor binding sites from both MmImpα and NcImpα are fully in agreement with the affinity assays (next section).

### Calorimetric assays for the binding of NIT-2 and PAC-3 NLSs and MmImpα

**Figure 5:**
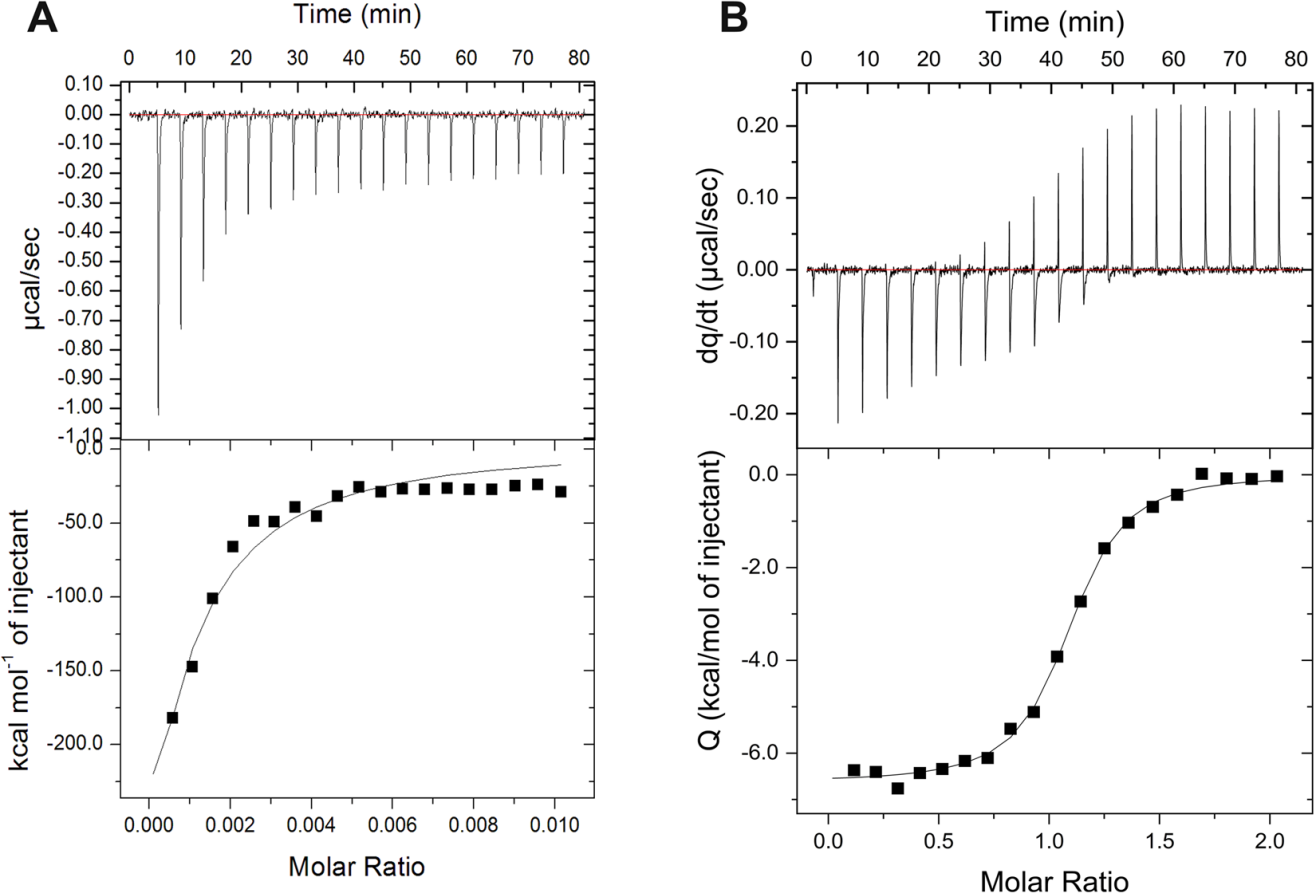
Isothermal calorimetric titration of NIT-2 and PAC-3 NLS peptides into MmImpα. The superior panel shows the raw data thermogram (thermal power as a function of time) of the titration of Impα with (**A**) NIT-2 and (**B**) PAC-3 NLS. The inferior panel shows the binding isotherm (ligand-normalized integrated heat as a function of the molar ratio). The data were determined by a general nonlinear regression model considering two ligand binding sites (solid line) or one ligand binding site.

Representative thermograms of calorimetric titrations for both complexes are shown in Fig. 5. Binding isotherms for NIT-2 and PAC-3 NLS peptides and the Impα receptor were best fitted with a nonlinear regression model of two nonidentical and independent binding sites or one binding site. The data processing revealed that two NIT-2 NLS peptides bind to Impα, but only one PAC-3 NLS peptide binds to Impα. For NIT-2 NLS, the dissociation constant (K_d_) was in the submicromolar range (~0.1 mM) and attributed to the major binding site, and the other constant corresponding to a 10-fold lower affinity was attributed to the minor binding site. In the case of the PAC-3 NLS, the K_d_ for the only binding site was also in the submicromolar range. Enthalpic parameters (ΔH) for all assays showed favorable enthalpic values: −8.09 ± 0.29 (NIT-2 NLS, major binding), −3.74 ± 0.14 (PAC-3 NLS) and −0,44 ± 0,19 (NIT-2 NLS, minor binding).

In addition, aiming to further understand the binding of the PAC-3 NLS to MmImpα, two mutated peptides (N and C-termini mutated basic clusters) were tested by ITC using the same experimental conditions employed by PAC-3 NLS: i) ^281^FDA**AAA**QFDDLNDFFGSVKRRQIN^304^ and ii) ^281^FDARKRQFDDLNDFFGSV**AAA**QIN^304^. ITC assays revealed that both mutated peptides present no measured interaction with Impα receptor (Suppl. Fig. 1), showing that the presence of both basic clusters are necessary for the PAC-3 NLS binding to Impα.

The comparison between MmImpα/NIT-2 NLS and NcImpα/NIT-2 NLS^51^ calorimetric assays reveals that K_d_ values for the major binding site are exactly the same and thus are compatible with the conservation of residue interactions (Fig. 3). The same comparison for the minor binding site reveals that NIT-2 NLS has a higher affinity for MmImpα/NIT-2 NLS, which is also compatible with the higher number of interactions observed in the MmImpα/NIT-2 NLS structure compared to the NcImpα/NIT-2 NLS structure (Fig. 3). The comparison between MmImpα/PAC-3 NLS and NcImpα/PAC-3 NLS^52^ calorimetric assays reveals that their K_d_ values are the same considering the experimental error.

## Discussion

### Comparison of monopartite NLSs binding to mammalian and fungal Impα

More than 120 crystal structures of Impα have been solved since 1998 (*S. cerevisiae* Impα, PDB ID 1BK5^37^) followed by the first mammalian Impα (*M. musculus*, PDB ID 1IAL^22^) and cocrystallized Impα with NLS peptides^7,36^. Most of the Impα structures deposited in the Protein Data Bank are MmImpα complexed to NLS peptides from several organisms^10^ but also synthetic NLS peptides^8^ and small molecules^57^. In addition, *H. sapiens* Impα variants^12–14,16,48,57^, *O. sativa*^33,39^, *A. thaliana*^40^ and *N. crassa*^41^ structures were also solved. The analysis of these structures clearly demonstrates that the overall Impα structures are highly conserved among them^8,41^, and only their solenoid curvatures may vary, particularly between proteins from different phylogenetic families^58^. However, few structural studies have compared different Impα variants from the same organism or Impα of different organisms.

A study with different human Impα variants complexed to influenza A PB2 NLS^15^ identified important differences among the variants: the Impα3 variant is more flexible than other variants; the Impα1 variant has the strongest autoinhibition and Impα 3 has the weakest inhibition. Two comparative studies between Impα from different organisms have also been performed^33,39,41^. OsImpα was solved complexed to the prototypical monopartite NLS from SV40 and with two synthetic “plant-specific” NLSs^41^. NcImpα was also solved complexed to SV40 NLS. Interestingly, the binding of the SV40 NLS to the major-binding sites from OsImpα, NcImpα and MmImpα were very similar^33,39,41^. The binding to the minor-binding site is shifted one position for OsImpα and NcImpα compared to MmImpα and presents some different interactions, particularly for the N- and C-termini of the peptide. Structural comparison and multiple alignment of Impα proteins show that some residues of the region near the minor site (Armadillo repeats 8 and 9) present in NcImpα (S402, E493 and K497), OsImpα (S394, E480 and K484) are not conserved in MmImpα (T402, S483, A487). These substitutions may prevent the binding of particular residues of NLS peptides to an Impα by steric hindrance or may cause different interactions of a particular peptide with different Impα proteins.

The structural conformation of SV40 and NIT-2 NLS peptides for MmImpα^41^ and NcImpα receptors are reasonably similar (P2-P5 positions), which is consistent with other monopartite NLSs^7,25,27,31,32,35,53,56^ (Fig. 4). In addition, for the major binding site, the interactions for both peptides and receptors are conserved (Fig. 3). However, for the minor binding site, different interactions occur for the different receptors and NLS peptides (Fig. 3). These differences are related to specific sequential differences between both receptors, as previously reported^33,39,41^, but also with the shifted position of the SV40 NLS bound to MmImpα^7^ that also present alternative binding modes to this site^7,24^. These alternative binding modes are likely related to the high content of sequential K/R residues of the SV40 NLS. The calorimetric assays described in this report (Table 2) are in agreement with the structural studies of these four complexes (NcImpα/NIT-2 NLS, MmImpα/NIT-2 NLS, NcImpα/SV40 NLS, MmImpα/SV40 NLS). The K_d_ values are on the same order of magnitude for the major binding site (0.56, 0.56, 0.89, and 1.8 µM for the same complexes) and present a higher variation for the minor binding site (9.9, 5.7, 1.7, 23 µM).

**Table 2:** Thermodynamic constants of Impα/NLS complexes interactions. Data obtained by ITC assays.

Complex	Stoichiometry	Kd (μM)	ΔH (kcal/mol)	ΔS (cal/mol/deg)
MmImpα/PAC3	1.06 ± 0.01	0.44 ± 0.05	6.63 ± 0.66	6.47
MmImpα/PAC3Δminor	No binding	—	—	—
MmImpα/PAC3Δmajor	No binding	—	—	—
NcImpα/PAC3^52^	1.02 ± 0.01	0.39 ± 0.07	−12.17 ± 0.17	−12.20
MmImpα/NIT2	0.95 ± 0.01	0.56 ± 0.23	−0.44 ± 0.19	−0.02
	0.95 ± 0.01	5.74 ± 0.99	−8.09 ± 0.29	−0.40
NcImpα/NIT2^51^	1.00 ± 0.01	0.56 ± 0.32	−6.64 ± 0.27	0.24
	1.00 ± 0.01	9.90 ± 1.10	−7.04 ± 0.20	4.58

**Table 3:** Binding of nuclear localization sequences to specific binding clusters in *Mus musculus* importin-α.

Protein		Minor NLS binding site						Linker		Major NLS binding site							Linker	PDB ID
		P0′	P1′	P2′	P3′	P4′				P0	P1	P2	P3	P4	P5		(P3′-P1)	
*Bipartite NLS*																		
Npl	A	V	K	R	P	A		ATKKAG		Q	A	K	K	K	K	LD	10	1EJY,3UL1
Rb			K	R	S	A		EGSNPPK		P	L	K	K	L	R		11	1PJM
N1N2	R	K	K	R	K			TEEESPLKD		K	A	K	K	S	K		12	1PJN
mCBP80		M	S	R	R			RHSYENDGGQ		P	H	K	R	R	K	TS	13	3UKZ
yCBP80	N	R	K	R	R	G	D	*FDEDENYRDFRPR*		M	P	K	R	Q	R	IP	18	3UKY
yPRP20			K	R	T	*V*		*ATNGDASGAHRA*			K	K	M	S	K		15	4OIH
MAL		L	K	R	K...				...L	K	L	K	R	A	R	LA	34	3TPM
FEN1	S	A	K	R	K	E		PEPKGS		T	K	K	K	A	K	T	10	3UVU
Bimax1	P	R	K	R	P	L	EW	DEDEEP		P	R	K	R	K	R	LW	12	3UKW
Bimax2	RR	R	K	R	K	R	EW	DDDDDP		P	K	K	R	R	R	LD	12	3UKX
53BP1	S	G	K	R	K			LITSEEERSPA				K	R	G	K	S	11	6IU7
PB2	K	R	K	R	D	S		*SILTDS*Q	T	A	T	K	R	I	R	MA	12	2JDQ, 4UAE
PAC-3	*FDA*	*R*	K	R	*Q*	*F*		*DDLNDFFG*		S	V	K	R	R	Q	I	12	6P6E
*Monopartite NLS*																		
TPX2			K	R	K	H...					...V	K	M	I	K	L	42	3KND
SV40 TAg	P	K	K	K	R	K	V		P	P	K	K	K	R	K	V		1EJL
SV40-TAg CN	K	K	K	R	K	V			P	P	K	K	K	R	K	V		1Q1S,1Q1T
AR									G	A	R	K	L	K	K	LG		3BTR
PLSCR1											G	K	I	S	K	HW		1Y2A
dUTPase	P	S	K	R	A	R	P		AIS	P	S	K	R	A	R	PA		4MZ5,4MZ6
Ku80									GPT	A	K	K	L	K	T	E		3RZ9
Ku70									NEGS	G	S	K	R	P	K	VE		3RZX
BFDV										Y	R	R	R	R	R	Y		4HTV
CLIC4										V	A	K	K	Y	R	N		3OQS
A89										L	G	K	R	K	Y			4BA3
B54		G	K	R	K	R				L	G	K	R	K	R	H		2YNR
PepTM		K	K	R	R	E	A		P	F	K	K	K	R	R	EA		3L3Q
αIBB									DEQ	M	L	K	K	R	N	VS		1IAL
XPG1	S	L	K	R	K	R				S	L	K	R	K	R			5EKF
XPG2		R	K	R	K	T	R			Q	K	K	R	R	K	LR		5EKG
PLSCR4	S	I	I	R	K	W	N											3Q5U
Guα	SRG	Q	K	R	S	F	SKAFGQ				Q	K	R	S	F	S		3ZIN
A28		R	K	R	G	Y	SVAF				R	K	R	G	Y	S		3ZIO
A58		R	K	R	T	W	RDAF				R	K	R	T	W	R		3ZIP
B6	H	R	K	R	K	F	SDAF				R	K	R	K	F	S		3ZIQ
B141	RQ	R	K	R	K	W	SEAF				R	K	R	K	W	S		3ZIR
Nup50	M	A	K	R	V	A	EKELTD...											2C1M
NIT-2		S	K	R	Q	R	R			S	S	K	R	Q	R	S		6P6A

NIT-2 NLS peptide binds to MmImpα and NcImpα with similar conformations at major and minor NLS binding sites according to the MmImpα/NIT-2 NLS and NcImpα/NIT-2 NLS crystal structures. Indeed, the ITC assays are completely in agreement with the structural data, which for the major binding site, the K_d_ value is exactly the same for both proteins, and for the minor binding site, the K_d_ value is on the same order of magnitude. However, a deeper analysis of NIT-2 binding to minor binding sites of these receptors reveals interesting differences. The presence of additional interactions of NIT-2-NLS with MmImpα compared to NcImpα, particularly at positions P3′ and P4′, may explain the higher affinity of this peptide to MmImpα (Fig. 2). Interestingly, in contrast with a previous comparison of NcImpα and OsImpα with MmImpα^33,39,41^, in which nonconserved residues from these Impα interact differently with the N-terminus of NLS peptide, the present study observed different interactions in the C-terminal region of peptide. The NcImpα and MmImpα residues are conserved (D280, N283, R315, N319, E354) (Fig. 3)(Suppl. Fig. 2); thus, the different interactions for the same NLS peptide may be related to different structural concavities of both Impα, as observed for the high RMSD when both structures are superposed (subsection: Comparison between MmImpα structures and MmImpα/NIT-2 NLS and NcImpα/NIT-2 NLS structures**)**. Therefore, we suggest that both the N- and C-termini are able to confer specificity to particular NLS sequences that are able to bind at minor binding sites.

Thus, the structural and calorimetric study with MmImpα complexed to NIT-2 NLS revealed that this peptide binds as classical monopartite NLS (consensus sequence: KK/RX(K/R)^4^) to a mammalian Impα, similar to a fungal Impα^51^. The NIT-2 NLS peptide (^917^SS**KR**Q**R**^923^) interacts with high affinity to major binding sites  of both receptors with K_d_ of the same order of magnitude (~0.1 µM) compared to other classical monopartite NLSs with high affinity to MmImpα^56^. As other classical monopartite NLS^10,56^, the NIT-2 NLS peptide is also able to interact with the minor binding site one order of magnitude weaker than the major binding site (~1 µM). For the minor binding site, different interactions between a particular NLS with MmImpα and NcImpα are observed.

### What is the role of PAC-3 NLS in PAC-3 protein transport?

PAC-3 is a transcription factor that is translocated to the nucleus at alkaline pH stress in *N. crassa*^52^. Calorimetric assays with the putative PAC-3 NLS and NcImpα demonstrated that this NLS peptide has a strong affinity (0.39 µM, Table 2) to NcImpα with a stoichiometry of 1:1^52^. Taking into account the calorimetric results and that its sequence resembles a bipartite consensus sequence (KRX_10–12_K(K/R)X(K/R)), with the exception of the P5 position (K/R), the authors of this study hypothesized that this NLS region is responsible for the recognition of the PAC-3 transcription factor by Impα. Thus, these components may form a complex that permits PAC-3 to be translocated to the nucleus under specific conditions. However, the authors of this study were not able to crystallize this complex to obtain structural information to confirm this hypothesis.

In the present study, we used the same PAC-3 NLS peptide and performed equivalent calorimetric and crystallographic studies using MmImpα. The calorimetric study demonstrated that the PAC-3 NLS peptide interacts with MmImpα with approximately the K_d_ value considering the experimental error and with the same stoichiometry of 1:1. Furthermore, the calorimetric study with mutated N and C-termini basic clusters of PAC-3 NLSs and MmImpα revealed interesting results. As both mutated NLS peptides were not able to bind to the protein, it is possible to conclude that both clusters are necessary for the interaction between PAC-3 and MmImpα, thus PAC-3 is a bipartite NLS, as previously suggested in the study with PAC-3 and NcImpα^52^.

The crystal structure of the MmImpα/PAC-3 NLS complex revealed that two fragments of the PAC-3 NLS peptide bind to MmImpα. In the major NLS-binding site, seven peptide residues (^297^SVKRRQI^303^) were unambiguously observed bound at positions P0-P6 of MmImpα. However, no electron density was found in the linker region and, for the minor NLS-binding site, electron density was presented for five residue main chains, but only for three side chains (KRR). This sequence is not compatible with the expected sequence for the minor NLS-binding site ^285^KRQ^287^ (P1′-P3′), because the electron density in the position P3′ is compatible with Arg side chain. The presence of electron density for only three side chains in the minor NLS-binding site with higher B-factors compared to the entire protein indicates low affinity of this region to the protein or peptide staggering. Side chain electron densities for the positions P0′, P4′ and P5′ are typical for other bipartite or monopartite NLSs, such as NIT-2 NLS presented here (Fig. 1B) and in other previous studies^23,24,59^. Taking into account ITC assays with PAC-3 NLS and the previous structural studies with Impα, we suggest that peptide staggering is occurring with N-terminal sequence of the peptide (^281^FDA**RKRQ**F^288^). The presence of an Arg residue preceding the Lys residue and the absence of a basic residue after KR residues that would bind at the position P3′, may explain this phenomenon. NLS peptide staggering has been previous observed for other complexes, particularly for the SV40 TAg NLS^7^ which presents a basic residue preceding the KR residues.

The lack of electron density in the linker region for PAC-3 NLS is also an intriguing result obtained here. However, previous structural results with bipartite NLSs also presented this common characteristic, such as for CBP80^8^, PRP20^35^, and PB2^12^^,^^15^. Thus, some features seem to be important for the stabilization of bipartite NLSs, in addition to the two basic clusters of the consensus sequence, and may led to absence of specific contacts between the PAC-3 NLS linker and MmImpα (Table 3):

1. The presence of Pro residues in the linker region, particularly in the position preceding P2 (P-1, P0, P1), may confer rigidity to the linker (e.g., Bimax1^8^, Bimax2^8,14^, TERT^14^, CBP80^8^ and RB^24^) favoring this interaction.
2. Presence of Lys/Arg residues in positions preceding the P1′ and following the P2′ positions (e.g., Bimax1^8^, Bimax2^8,14^, CBP80^8^, FEN1^59^ and N1N2^23^) is also favorable.
3. Long length linkers are less favorable, as previously observed for the N1N2^23,53^; CBP80^8^, PRP20 and PB2^12^^,^^15^.
4. Polar residues in the linker region, as found for Bimax1^8^, Bimax2^8,14^, CBP80^8^ and N1N2^23^, also seem to be favorable.

Thus, the structural and calorimetric study with MmImpα complexed to *N. crassa* PAC-3 NLS revealed that this peptide binds to mammalian Impα. Considering that Impα structures are highly conserved^10^ and, particularly, that their major and minor NLS-binding sites are also strictly conserved, we suggest that the binding of PAC-3 NLS to MmImpα and NcImpα is similar. Indeed, the similarity of NIT-2 NLS binding to both MmImpα and NcImpα also supports this supposition. Thus, the present study confirms the hypothesis proposed by Virgilio and colleagues^52^ and enables us to understand the structural determinants for the interaction between the PAC-3 transcription factor and NcImpα and its translocation to the nucleus of this fungus.

## Conclusions

In the present work, NLSs from different *N. crassa* transcription factors (NIT-2 and PAC-3) were studied by structural and calorimetric techniques in complex with *M. musculus* Impα. The comparison of these data with previous results^51,52^ revealed remarkable similarity of the interaction between these sequences and *N. crassa* or *M. musculus* protein receptors. The NIT-2 NLS peptide binds as a classical monopartite NLS with high affinity to the Impα major binding site for both organisms. Calorimetric assays demonstrated that the PAC-3 NLS peptide interacts with Impα from both organisms with approximately the same affinity and stoichiometry indicating that it is a bipartite NLS. Since the main docking event occurs between the NIT-2 and PAC-3 NLSs and Impα at the major binding site, we hypothesized that the full-length NIT-2 and PAC-3 interact similarly with Impα from these two organisms.

The analyses of NIT-2 NLS minor binding sites of both Impα proteins reveal some particular interactions that corroborate the different affinity values obtained in this study. The higher affinity of *N. crassa* NIT-2 by MmImpα instead of NcImpα is an unexpected result, but strongly indicates that the major binding site is the site used for the translocation of NIT-2 protein to the nucleus. In contrast, the comparison between MmImpα/SV40 NLS and NcImpα/SV40 NLS revealed a higher affinity of the SV40 NLS for the minor binding site of NcImpα than for MmImpα^41^. A similar result was also observed for rice Impα^33^. In light of these results, we hypothesized that the differential affinity for NLSs at the minor site may be a useful strategy for organisms that only have one Impα isoform to selectively recognize and transport different NLSs.

## Experimental Procedures

### Protein expression and purification

The gene encoding the protein Impα from *M. musculus* was cloned into the pET30a expression vector. Recombinant MmImpα was cloned with a histidine tag and as a truncated protein (70–529) to avoid autoinhibition^22^. The clones were provided by Dr. Bostjan Kobe from the University of Queensland (Australia). The plasmid was expressed in *Escherichia coli* host strain Rosetta (TM) pLYS (Novagen), and the recombinant protein was purified by affinity chromatography according to Barros *et al*., 2012^59^. The protein was eluted with a 0.0–0.15 M imidazole linear gradient, concentrated using an Amicon dispositive, and the buffer was changed to 20 mM Tris-HCl, pH 8.0 and 100 mM NaCl for storage. The purified protein was stored at cryogenic temperature. NLS peptides NIT-2-NLS (^915^TISSKRQRRHSKS^927^) and PAC-3-NLS (^281^FDARKRQFDDLNDFFGSVKRRQIN^304^) were synthesized by GenOne with 98% purity.

### Isothermal titration calorimetry

MmImpα and NIT-2-NLS were diluted at 40 μM and 800 μM, respectively, in buffer containing 20 mM Tris-HCl, pH 8.0 and 100 mM NaCl. The samples were submitted to ITC experiments, performed with a MicroCal iTC200 microcalorimeter (GE Healthcare), where the peptide sample was titrated into the protein sample. The affinity data were obtained at 20 °C from 20 titrations of 2 μL, with 240 s of interval between each titration and 800 rpm homogenization speed. Experiments with MmImpα and PAC-3-NLS (native and mutated) were performed under similar conditions but with a protein/peptide proportion of 1:10. Further experiments with mutated PAC-3 NLS were also performed with a protein/peptide proportion of 1:20. Control experiments were performed by titration of the peptide sample into the buffer, and the data obtained were subtracted from the peptide:protein titrations. Data were processed using Origin 7.0 software (*Microcal Software, Northampton, MA*) to obtain the thermodynamic constants of the interactions^60^.

### Crystallization and structure solution

The complexes MmImpα/PAC-3-NLS and MmImpα/NIT-2-NLS were submitted to crystallization experiments using similar conditions as previous MmImpα/NLS peptide complexes^24,32,59^. Crystallization drops containing 1.0 μL of protein (18 mg/mL) 0.5 μL of peptide (5 mg/mL) and 0.5 μL of reservoir solution were mounted in hanging-drop plates and stored at 18 °C. Single crystals were obtained with reservoir solutions containing 0.55 M sodium citrate (pH 6), 1.6 M sodium citrate and 10 mM DTT after 7–14 days. Crystals obtained were submitted to X-ray diffraction at the Brazilian Synchrotron Light Source (LNLS) in Campinas-SP, Brazil. X-ray data collected were processed using XDS software^61^, and the structures were obtained by Fourier synthesis using MmImpα/Ku80-NLS as a template^32^ and refined using PHENIX^62^. Modeling of the peptides were performed using Coot^63^. All structural figures were generated using PyMOL v.1.8.6^64^ program.

## Supplementary information

Supplementary Information.

Supplementary Information 2.
