# Supplementary figures and images for: Comparative study of the interactions between fungal transcription factor nuclear localization sequences with mammalian and fungal importin-alpha
Source: Sci Rep. 2020 Jan 29;10:1458. doi: 10.1038/s41598-020-58316-9 (PMC6989684; doi:10.1038/s41598-020-58316-9)

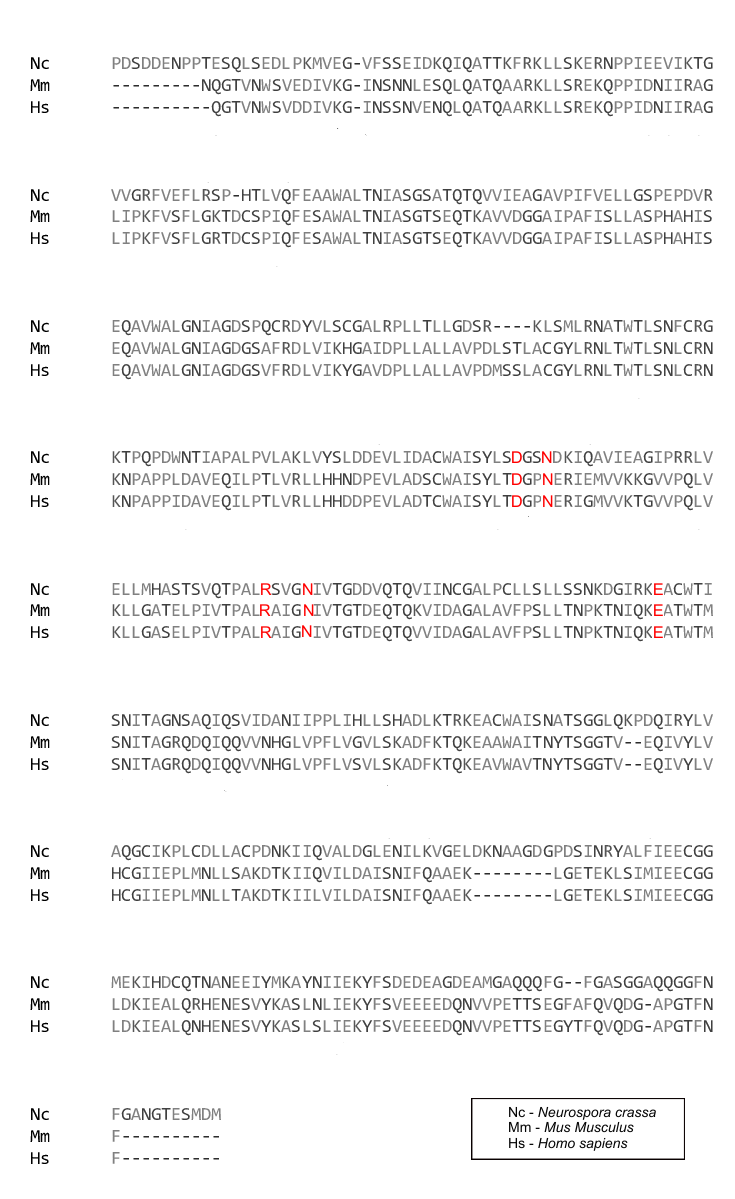

Supplement: Supplementary file 1 — Supplementary Information. [file 41598_2020_58316_MOESM1_ESM.tif]

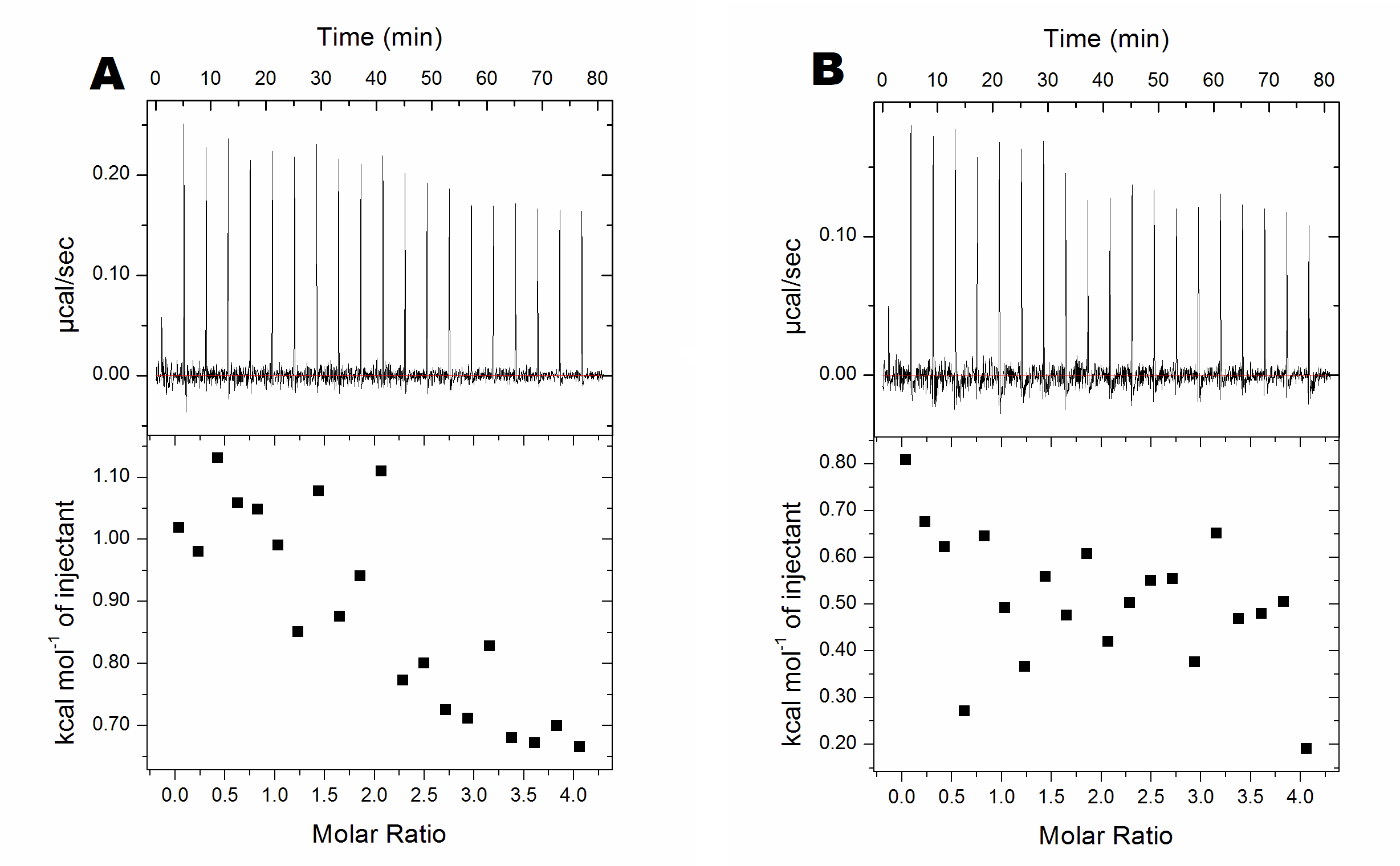

Supplement: Supplementary file 2 — Supplementary Information 2. [file 41598_2020_58316_MOESM2_ESM.tif]
